# Exploiting Mobile Edge Computing for Enhancing Vehicular Applications in Smart Cities

**DOI:** 10.3390/s19051073

**Published:** 2019-03-02

**Authors:** Hesham El-Sayed, Moumena Chaqfeh

**Affiliations:** College of Information Technology, UAE University, Al Ain 15551, UAE; moumena@uaeu.ac.ae

**Keywords:** mobile edge computing (MEC), intelligent transportation systems (ITS), smart cities, vehicular ad hoc networks (VANETs), vehicular edge computing (VEC)

## Abstract

Mobile edge computing (MEC) has been recently proposed to bring computing capabilities closer to mobile endpoints, with the aim of providing low latency and real-time access to network information via applications and services. Several attempts have been made to integrate MEC in intelligent transportation systems (ITS), including new architectures, communication frameworks, deployment strategies and applications. In this paper, we explore existing architecture proposals for integrating MEC in vehicular environments, which would allow the evolution of the next generation ITS in smart cities. Moreover, we classify the desired applications into four major categories. We rely on a MEC architecture with three layers to propose a data dissemination protocol, which can be utilized by traffic safety and travel convenience applications in vehicular networks. Furthermore, we provide a simulation-based prototype to evaluate the performance of our protocol. Simulation results show that our proposed protocol can significantly improve the performance of data dissemination in terms of data delivery, communication overhead and delay. In addition, we highlight challenges and open issues to integrate MEC in vehicular networking environments for further research.

## 1. Introduction

The major objective of intelligent transportation systems (ITS) is to improve traffic safety, transportation efficiency and driving experiences. Despite the fact that vehicular ad hoc networks (VANETs) have been viewed as one of the most enabling technologies for connected vehicles in the context of ITS, huge amounts of traffic data bring serious challenges to VANETs due to issues correlated with connectivity, scalability, flexibility and intelligence [1]. To take the advantages of cloud computing in serving VANETs with computational services, vehicular cloud computing (VCC) was introduced [2]. The main objective of integrating cloud computing in vehicular environments is to provide dynamic applications that can predict traffic events and adapt to environmental changes.

VCC has greatly improved resource utilization and computation performance in vehicular environments, however, capacity limitation and networking transmission delays are being considered as serious issues, especially when the cloud servers are far away from the vehicles on the road. To bring computing capabilities and IT service environment closer to endpoints, mobile edge computing (MEC) has been recently introduced [3]. MEC provides low latency responses, high bandwidth, and real-time access to network information via applications and services. These characteristics enable MEC to offer an ideal platform for vehicular networks, which are highly dynamic environments that essentially require supporting real-time data. Due to the characteristic of proximity, MEC environment can provide fast responses in the context of connected vehicles, which are currently being anticipated to replace smartphones as the computing devices of the next decade [4].

Several attempts have been made to integrate MEC in the context of ITS, including new architectures [1,5,6], communication frameworks [5], deployment strategies [7] and applications [1,8,9]. Nevertheless, to the best of our knowledge, none of the existing work has presented a detailed implementation for integrating MEC at the application level and characterizing its performance impact. Taking this gap into account, this paper mainly aims to serve as a bridge for researchers who plan to move their proposed solutions from conventional VANETs in the direction of MEC. To achieve our main objective, we propose an edge-based data dissemination protocol which addresses efficiency and scalability in vehicular networks. Efficiency in this work refers to providing full coverage without extra communication overhead, whereas scalability refers to maintaining low data redundancy and delay under different traffic conditions.

Existing data dissemination protocols often aim to reduce data redundancy. For an improved performance, this reduction should consider the number of vehicles on the road (which we refer to as traffic density) to control the selection of forwarding vehicles. This is due to the fact that selecting more forwarders than necessary would dramatically increase data redundancy and waste the limited channel bandwidth. To provide a traffic density estimation, the majority of existing protocols exploit beaconing with a fixed period [10,11,12,13,14,15,16,17,18,19], which has several drawbacks on the networking performance, such as wasted bandwidth and increased network congestion [20]. Other protocols which do not rely on beacons usually ignore the condition of the traffic, and therefore, they do not scale well under high-density scenarios [21,22,23].

In contrast, our edge-based protocol exploits an EC architecture for traffic condition estimation. This estimation is utilized in controlling data dissemination for an improved efficiency and scalability. In summary, the contributions of this work are three fold:

- First, we provide a review of the current efforts to integrate MEC in vehicular environments, with a classification of potential applications and a description of the associated challenges and issues for further research.
- Second, we propose an edge-based data dissemination protocol which addresses efficiency and scalability in vehicular networking environments. Our protocol accommodates the two major classes of vehicular networking applications: traffic safety and travel convenience.
- Third, we provide a simulation-based implementation for evaluating our protocol at the application level in comparison with existing approaches.

The remainder of this paper is organized as follows: a literature review on integrating MEC in vehicular environments is presented in Section 2. In Section 3, we classify the potential applications. In Section 4, we describe our proposed protocol, whereas in Section 5, we evaluate its performance via simulation results. In Section 6, we highlight the associated challenges and issues. The last section concludes the paper.

## 2. Literature Review

VANETs were firstly introduced in 2001 for providing vehicular communication systems and networking applications [24,25]. VANETs are then seen as a key component of ITS. In the last two decades, VANETs have been heavily researched as one of the most enabling technologies for connected vehicles [26,27]. However, the current VANET architecture suffers from miscellaneous issues correlated with connectivity, computation, scalability, flexibility and intelligence [1].

Vehicular cloud computing (VCC) was introduced [2] to take the advantages of cloud computing in serving VANETs with computational services. The main objective of integrating cloud computing in vehicular environments is to provide dynamic applications that can predict traffic events and adapt to environmental changes. Despite that VCC can greatly improve resource utilization and computation performance in vehicular environments, capacity limitation and transmission delays are considered as serious issues when the cloud servers are far away from the traveling vehicles. More specifically, big traffic data poses a major challenge for the cloud computing paradigm due to low transportation speed. For example, when autonomous vehicles are on the road, each vehicle is expected to generate 1 GB data every second [28]. Instead of directly connecting to the cloud for sending data, vehicles can utilize real-time processing in an edge computing environment for an improved performance in terms of response time, computation efficiency and resources utilization.

The characteristics of edge computing include low latency and location awareness, wide-spread geographical distribution, mobility, heterogeneity, scalability, the predominant role of wireless access and the strong presence of real-time applications [29]. These characteristics make the edge the appropriate platform for a number of critical Internet of Things (IoT) services and applications, namely, connected vehicle, smart grid, smart cities, and in wireless sensors and actuators networks. In addition, the edge will give rise to new forms of competition and cooperation between providers angling to offer global services [29]. A general survey on edge computing can be found in [3].

Edge computing refers to “the enabling technologies allowing computation to be performed at the edge of the network, on downstream data on behalf of cloud services, and on upstream data on behalf of IoT services” [28]. The term “edge” may refer to any computing resource between data sources and the cloud. In a vehicular environment, a smart traffic light can represent an edge between the vehicles in its coverage and the cloud data center. To clearly understand the difference between conventional cloud computing and the edge computing paradigm, we encourage the reader to refer to [28].

In the context of vehicular environments, vehicles can directly connect with edges for data collection and service provision, instead of directly connecting to the cloud. As a significant part of IoT, vehicular communication systems would produce and consume data at the edge for an improved performance. In our literature review, we found it promising to integrate edge computing into vehicular networks for a vehicular edge computing (VEC) environment. An example framework for VEC can be found in [30], which aims to increase the computational capabilities of vehicles on the road. It introduces a workflow to support the autonomous organization of edges in vehicular environments. In the remainder of this section, we describe VEC enabling technology, proposed architectures, deployment strategies and potential applications.

### 2.1. Enabling Technology

Two networking paradigms can be considered for the integration of VEC: 5G and software defined networking (SDN) [1]. 5G can offer better response times, greater coverage and more efficient signaling. On the other hand, SDN provides flexibility, scalability and programmability, by separating the data plane from the control plane. This way, network development, deployment and management are simplified [31]. The communication components to consider for VEC are SDN global controller, 5G base station (BS), SDN roadside units (RSUs) and SDN wireless nodes [1]. In a recent work [32], the authors investigate practical deployment technologies and promising technical enablers for VEC, including SDN, network function virtualization (NFV), smart collaborative networking (SCN) and blockchain. They proposed a collaborative VEC framework, which can support scalability in vehicular networks at the application level. In another recent work [33], the distribution of content in the context of 5G edge-based vehicular networks is investigated.

### 2.2. Architecture

Different architectures have been recently proposed for the implementation of edge computing in vehicular environments [1,5,6,31]. The authors in [5] tried to address latency in the internet of vehicles (IoV), by integrating edge computing and SDN. Edge computing is utilized to offer a highly virtualized platform that provides computation, storage, and networking services between end devices and traditional cloud computing data centers. The network architecture is divided into four layers: cloud computing layer, SDN control layer, edge computing layer and infrastructure layer.

**Figure 1:**
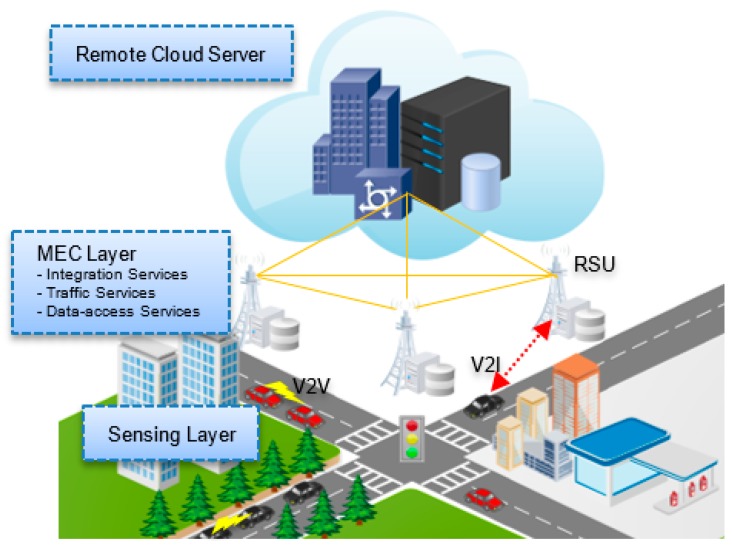
The VEC Architecture.

Similarly, the authors in [1] proposed a four-tier architecture for urban traffic management with the convergence of VANETs, 5G networks, SDNs, and mobile edge computing technologies. The authors used a paradigm of road accident rescue to validate the high efficiency of the proposed architecture. Three measures were proposed to improve the rescue system. These are remote video diagnosis and initial assessment, early-warning and no-go area, and green rescue lane for emergency vehicles. The proposed architecture may decrease traffic congestion and improve the ability to manage urban traffic for faster rescue. In the following, we alter the architecture provided in [1] for a generalized VEC architecture that consists of three layers: the traffic sensing, the mobile edge computing and the cloud server layer as shown in Figure 1. These layers are briefly described below:

#### 2.2.1. The Traffic Sensing Layer

This layer combines two types of entities in a hierarchical architecture: a large number of mobile vehicles with limited computation and communication capabilities, and a set of roadside units (RSUs). Each entity has its own local environmental view. Each RSU is equipped with a processing unit for collecting data within its transmission range. Specifically, RSUs sense passive data within their coverage including the number of vehicles and license plate numbers. On the other hand, vehicles act as a source of active data including speed, direction and location information.

#### 2.2.2. The Mobile Edge Computing Layer

To introduce network edges in a MEC layer, some of RSUs can be equipped with mobile edge computing (MEC) server units. MEC servers can also be deployed at temporal architecture components such as drones. In general, any entity that is equipped with a MEC unit would play the role of a network edge to offer various services including integration, localization, traffic data processing, traffic condition detection, connectivity and data access services with the aid of the cloud server layer. Vehicles can directly communicate with nearby network edges for service provision and data collection.

#### 2.2.3. The Remote Cloud Server Layer

This layer presents high computational power and storage capabilities of traffic data in addition to a wide range of services and applications. Network access can be provided on-demand to network edges and mobile vehicles with an Internet connection. To facilitate urban traffic management, learning algorithms can be employed to offer driver convenience services such as travel time estimation, congestion detection and route guidance.

### 2.3. Communication

Communication management is essential among vehicles and network edges, and among the distributed edges themselves. The focus on the provision of communication management systems in the context of a MEC environment is still limited. In [9], the authors proposed a publish-subscribe semantic-based MEC architecture to enable interactions at the semantic level among geo-distributed edge nodes. It assumes that a publisher issues a message with a topic, and defines the class of an event pattern to which a subscribing edge node can subscribe. Among receiving edges, only interested nodes (subscribers) accept the message, and then cooperate in responding to it. A proposed domain ontology is utilized to define related concepts and relationships among them. In addition, an event-driven architecture based on active rules is used to exchange semantic resources among edges. Specifically, an active rule defines a set of actions to take in response to a specific semantic event based on the ontology. It uses a well-defined formalization based on a structure of event-condition-action rules. An event matcher captures semantic events by evaluating resources with a set of active rules, and a condition evaluator checks if a specific action can be taken upon event detection. A sequence of actions is managed by an action scheduler, and semantic events are forwarded by a knowledge propagator to interested nodes or to the cloud.

A considerable research is focusing on load balancing in an edge-based vehicular network. In [5], the authors aim at achieving optimal load balancing, by utilizing constrained optimization particle swarm optimization algorithm. When a task is transferred from end users to the edge, it is divided into several subtasks. A subtask may be transferred to the fog node or cloud node for calculation. The node which distributes a task will also execute part of it. Results are finally merged and sent back to the end users. The problem is modeled as minimizing the maximum latency of subtasks over the network, and simulation results prove the efficiency of the proposed algorithm in latency reduction.

### 2.4. Deployment

The deployment of network edges within city environment is a multi-factor dependent problem, since it relies on static components such as roads layout, segments length, and the number of lanes, in addition to dynamic factors including mobility patterns, driving behaviors, accident frequently occurring areas and vehicles distribution [7]. Fixed RSUs can be equipped with edge computing capabilities to act as network edges and provide edge-based services. However, the employment of flying RSUs that are carried by unmanned aerial vehicles (UAVs) would enable supporting traffic safety and convenience applications dynamically in accordance with traffic events and congestion conditions. The key benefit of this dynamism is to enable edge computing environment with a constrained budget.

Instead of equipping fixed RSUs with MEC servers, UAVs can be utilized to present mobile network edges. Many new ITS applications could be enabled by realizing UAVs to improve traffic safety and enhance travel comfort. Before being able to utilize UAVs in such applications, there are some serious issues to address including limited energy, processing capabilities, and signal transmission range. Taking into account the technology revolution in the past decades, there is a great potential that these limitations in UAV utilization will be resolved in the near future. Challenges to enable UAVs in the next-generation ITS are described in [34].

## 3. Potential Applications

A number of characteristics are enabling the network edge to present an ideal platform for providing a rich set of applications and services in the context of vehicular environments. These characteristics are geo-distribution, mobility and location awareness, low latency, heterogeneity, and support for real-time interactions [29]. In contrast with VANET, and from our point of view, VEC has the following potential benefits to ITS:

- Connectivity: vehicles with no Internet connection can be supported with safety and convenience services on demand, by providing timely information through direct communication between the requesting vehicles and the nearest edge. In safety conditions, edges broadcast warning messages to vehicles within their coverage area. Moreover, vehicles can request the nearest edge for convenience service provision. The edge can connect to the Internet if necessary and provide a response to the requesting vehicles.
- Traffic Monitoring: Traffic events can be effectively detected by network edges with high accuracy according to monitored traffic conditions. Vehicles can be warned of a potential crash or a predicted congestion to take proper actions.
- Dissemination Efficiency: Due to the characteristics of proximity and communication coverage, network edges can provide efficient data dissemination with high delivery ratio and acceptable latency to relatively faraway distances.
- Trust Management: Edges have the potential to generate trusted safety and convenience messages in a context of trust management system, which can address many of the existing trust management in VANET.
- Enhanced privacy; since user data can be produced and consumed locally and anonymously without the need to store it or upload it to the cloud.

In the remaining of this section, we review existing efforts to push computing out onto the edge in the context of vehicular environments. Potential applications include data collection and analysis, vehicular control systems and traffic management.

### 3.1. Data Collection, Dissemination and Analysis

Data collection in vehicular environments aims at supporting real-time interactions for an improved safety and convenience. Smart traffic lights (STLs) illustrate an example of real-time interactions support. STL can directly interact with nearby sensors for pedestrian detection, speed monitoring and location data collection. A set of cooperating STLs can play a key role in traffic management by coordinating the green traffic wave [29]. Moreover, equipping STLs with edge computing capabilities can effectively contribute to predicting potential crashes, with the aid of real-time data analysis and learning algorithms. Consequently, approaching vehicles can be warned in advance to take proper actions. The collected data can be aggregated and summary values can be sent to the Cloud for global, long-term analytics.

An example application that benefits from edge computing can be found in [9], where edge nodes continuously collect traffic data from nearby vehicles and STLs. Collected data is processed in two layers for acquiring high-level knowledge. At the first layer, the immediate processer captures various event patterns from the collected data. For example, when an edge node identifies an accident event from an analysis of a crashed car data, it notifies other edges in addition to vehicles nearby the accident location. The second layer contains processing components to derive high-level knowledge from the data processed at the first layer.

### 3.2. Vehicular Control Systems

Edge computing can address challenges in control systems of autonomous vehicles, which are: limited sensing areas, the possibility of deadlock, and the expensive cost of the vehicle equipped with rich sensors. In [8], the authors proposed a cloud-based vehicle control system that periodically aggregates sensor data from multiple vehicles in the vicinity and controls them from the cloud. And edge server is used as a computational node together with the cloud-based vehicle control system. Both the cloud and edge servers have vehicle controllers and are referred to as the cloud controller and the edge controller. To balance the computational load between the edge and cloud servers, an automatic switching method of vehicle controller between the edge and cloud servers is proposed. For performance evaluation, the authors implement an interesting prototype system using microcars and evaluate the stability of the driving trajectory, the control ratio, and the amount of traffic.

### 3.3. Traffic Management

Traffic management systems can play a key role in the context of smart cities for safer and more convenient roads. Providing traffic management solutions to decrease traffic congestion level is crucial for rescue systems efficiency. The authors in [1] proposed to provide a rescue system to decrease traffic congestion and improve the ability to manage urban traffic. They described a case study of rapid road accident rescue. The verification of the proposed system was simplified by selecting 10 accidents randomly from an accidents dataset in a month, and then using the data to simulate the practical traffic flow. Two assumptions were made: 60% of drivers keep clear of an ambulance without an electronic police monitor, and 95% of them keep clear of an ambulance with a police monitor. They show a preliminary validation of the system in normal times and in rush hour. Firstly, they compare three solutions in normal times without congestion. Secondly, they compare them during rush hour. Their solution saved 9.9 min without a police monitor and 12.5 min with a police monitor in the first-arrival time of ambulance prospect, and saved 15.5 and 19.5 min respectively in traffic recovery time.

### 3.4. Trust Management

A major objective of vehicular networking is to improve road safety and reduce traffic congestion. The experience of individual vehicles on traffic conditions and travel situations can by shared with other vehicles for improving their route planning and driving decisions. To avoid making inappropriate decisions, it is essential to ensure the trustworthiness of other vehicles which may behave maliciously by injecting false information in the network. An efficient trust management system should accurately identify the trustworthiness of individual vehicles and detect malicious drivers. Existing trust management solutions in this context follow either the centralized cloud-based approach, or the fully decentralized P2P approach [35]. Each of these approaches has its own drawbacks that eliminate addressing the requirements and challenges of vehicular networking applications and services.

An edge computing architecture can overcome the limitations of both approaches, by providing low-latency, high scalability and efficient data access services. Despite its essential role, trust management research in the context of edge computing is quite limited, basically because it is a very recent paradigm, and its infrastructure is not standardized yet [36].

## 4. The Proposed Protocol

In this section, we describe our MEC protocol for efficient data dissemination in vehicular networks. Our protocol supports the two major classes of vehicular networking applications, which are traffic safety and travel convenience. Traffic safety applications require high data coverage with low latencies, whereas travel convenience applications require efficient data delivery and retrieval. These applications are often referred to as delay-tolerant applications. Examples include travel time estimation, speed expectation and congestion detection.

### 4.1. A Protocol Overview

Our proposed protocol consists of two major components: efficient traffic data representation and delay-controlled data dissemination. For efficient data representation, network edges are proposed to monitor the traffic for indicating events and conditions. These conditions are shared among vehicles through a delay-controlled data dissemination component. Network edges are involved in the dissemination of safety and convenience messages in a vehicular environment for an improved performance, connectivity and trust. By integrating edges, data can be disseminated with high delivery ratio and low latency to relatively faraway distances. Such a performance enables the edge to provide an ideal platform to fulfill the requirements of different types of vehicular networking applications. In addition, vehicles with no internet access can be warned of a potential crash by connecting to the nearest edge, even if VANET is sparsely connected. To overcome the limited computation power of the edge, decisions can be made with the aid of the vehicular cloud when necessary.

In the remaining of this section, we describe the problem which we are proposing a solution for. Then, we explain the role of edges in traffic condition detection and representation. After that, we provide a description of how vehicles control their broadcasting delays according to the traffic condition for efficient data dissemination. Lastly, we explain how our protocol can be integrated into delay-tolerant applications.

### 4.2. Problem Description

The majority of vehicular networking applications rely on broadcasting to disseminate safety and convenience data, which can easily lead to the broadcast storm problem under high-density scenarios. The broadcast storm problem occurs when many vehicles in the same vicinity broadcast nearly simultaneously. This phenomenon leads to a high overhead of redundant data, in addition to data loss or message corruption due to collisions. Therefore, simple broadcast does not scale in dense networks due to the excessive dissemination of the same data, which wastes the limited available channel bandwidth. In a recent work [37], the authors proposed interest-aware data dissemination protocol for delay-tolerant vehicular networks, which combines probabilistic broadcast and timer-based broadcast. Their simulation results showed that the proposed protocol costs less than simple schemes in obtaining a high data delivery, however, the overhead performance was not measured.

To eliminate unnecessary transmissions for the purpose of redundancy overhead reduction, existing data dissemination protocols often provide a mean of broadcast control, by selecting a subset of vehicles to relay data further. The majority of these protocols rely on a fully distributed V2V architecture for estimating the number of vehicles nearby [10,11,12,13,14,15,16,17,18,19], which we refer to as *traffic density*. This estimation is utilized to select relays at each hop based on the traffic condition until the area of interest is fully covered. To do so, existing protocols exploit beaconing with a fixed period, which has several drawbacks on the networking performance, such as wasted bandwidth and increased network congestion. For instance, when vehicles send a 200-byte beacon every 100 milliseconds, the channel would be 80% loaded at the range of 300 m [20]. Other protocols which do not rely on beacons usually ignore the condition of the traffic [21,22,23], and therefore, they do not scale well under high-density scenarios.

In this work, we aim to propose and evaluate an efficient data dissemination protocol that addresses scalability in vehicular networks without relying on beacons. Efficiency in this work refers to providing full coverage without extra communication overhead, whereas scalability refers to maintaining low data redundancy and delay as density increases. To this end, we rely on an edge-based traffic condition estimation by utilizing an EC architecture. This estimation enables our protocol to scale well under high-density scenarios. Due to the potential of the edge in detecting traffic conditions in all possible directions in urban areas, data dissemination can be effectively suppressed for a minimized data redundancy overhead, by setting the broadcast criteria according to the condition of traffic in each direction.

### 4.3. Traffic Condtion Detection and Representation

The detection of traffic condition may effectively benefit different types of applications correlated with congestion management and navigation systems. These systems can rely on the detection of traffic condition in different road segments for an enhanced routing recommendations. In our protocol, we propose to utilize traffic condition information in controlling the dissemination of safety messages to provide high data delivery without the drawbacks of unnecessary transmissions. More specifically, excessively broadcasted messages under high density traffic scenario may easily result in packet loss due to collisions, however, providing an efficient broadcast suppression according to traffic conditions is the solution we propose for an improved data dissemination in vehicular networks.

Instead of relying on local data for detecting traffic condition as we have proposed in our previous work [38], we propose to utilize a MEC architecture for accurately detecting the condition of any road segment in the network. Data dissemination can then be controlled via both V2I and V2V communication. The network is represented as a directed graph *G* = (*V*, *E*) where the set of *n* vertices *V* = {*v*_1_, *v*_2_, *v*_3_, …, *v_n_*} represents road intersections, and the set of *m* edges *E* = {*e*_1_, *e*_2_, *e*_3_, …, *e_m_*} represents the links between these vertices, where *E* ⊆ {(*u*, *v*)|*u*, *v* ∈ *V* ∧ *u* ≠ *v*}. To indicate traffic condition of each road link in the network, an irreflexive weighted adjacency *n* × *n* matrix *A* is defined as follows:

(1) $$A={\left[w_{ij}\right]}_{t_{x}}=\{\begin{array}{cc} {\left(CF_{ij}\right)}_{t_{x}}, & if\;a\;link\;from\;i\;to\;j\;exists\\ 0, & otherwise \end{array}$$

where *w_ij_* is the weight given to the link connecting *v_i_* and *v_j_* according to its traffic condition at time *t_x_*. We define the traffic Condition Factor (*CF*) which combines a set of weighted parameters to indicate traffic condition of a road link during a predefined time interval. In addition to active parameters such as speed, acceleration and direction, *CF* can include parameters inferred from historical data such as accident frequency. For a set of *n* parameters with defined weighs, the condition factor of a road link connecting *v_i_* to *v_j_* is computed as:

(2) $$CF_{ij}=\overset{n}{\underset{k=1}{\sum }}{\left(P_{k}.w_{k}\right)}_{ij}$$

where *P_n_* is the set of weighted parameters utilized in calculating *CF*, and *w_n_* is the set of weights given to them. The weighted matrix is updated whenever a new weight is calculated. The cloud server keeps a record of calculated weights for long-term data analysis. A network edge needs only to keep the last updated weights of the road links belong to the area it supervises. Edges can share their weights with nearby vehicles and edges or request for information about missing weights depending on the application.

Active data generated by mobile vehicles are collected by the nearest edge. Here we focus on the speed as an example active parameter to consider for calculating *CF* for two main reasons: simplicity and effective representation. Specifically, the speed-density relationship is the most descriptive of driver behavior, since average speeds decline as density increases. In other words, traffic density can be derived from the speed and flow rate, which are relatively simple traffic measurements. Therefore, speed data series generated by mobile vehicles is a representative collection of data that can be utilized by network edges not only to estimate traffic condition, but also to derive other parameters. If speed is the only considered parameter, then:

(3) $${\left(CF_{ij}\right)}_{t_{x}}=\frac{1}{t_{x}-t_{0}}\overset{t_{x}}{\underset{t=t_{0}}{\sum }}\frac{\bar{V_{t}}}{V_{f}}$$

where ${\bar{V}}_{t}$ is the mean speed computed during time interval [*t*_0_, *t_x_*] and *V_f_* is the free-flow speed, which is utilized to weight the speed parameter. In [39], we show that the participation of low percentage of vehicles can be sufficient to indicate traffic condition.

**Figure 2:**
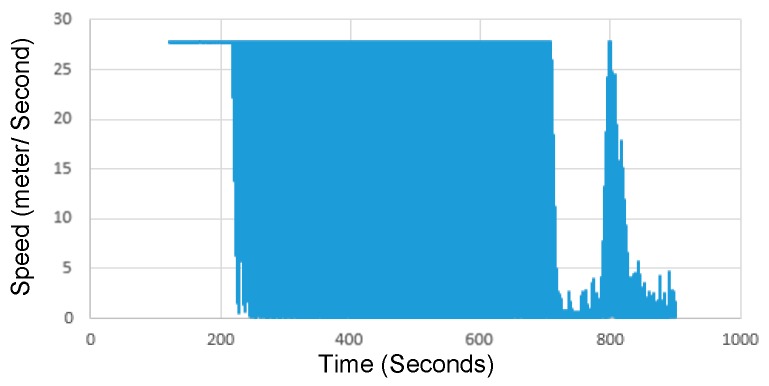
Speed data of a sample road generated by individual vehicles and collected by the nearest edge.

Individual vehicles are expected to record their own speed data and share them with the nearest edge. We assume that edges are integrated into a subset of RSUs. The edge computes a resultant speed graph for each link in its transmission range from the collected data, by computing the average speed at each time interval. In Figure 2, we show a sample speed data of a road link collected from the traversing vehicles by the nearest edge. This sample is extracted from the results generated by simulating the traffic scenario described in Section 5. This sample data illustrates how speed parameter is effectively representative, because it enables not only traffic condition estimation, but also defining congestion points. As the figure shows, vehicles start to accelerate until reaching the maximum allowed speed, where a free-flow condition can be estimated. After around 700 s, vehicles start to travel at low speeds, where traffic congestion can be indicated. Around 100 s later, the congestion is dissolved, before starting to form again.

Network edges are responsible for calculating the weights of the links they supervise, and then store them in their local databases. Edge nodes have to communicate with the remote cloud server to upload data or answer decision making queries based on comprehensive data analysis.

### 4.4. Delay-Controlled Data Dissemination

**Figure 3:**
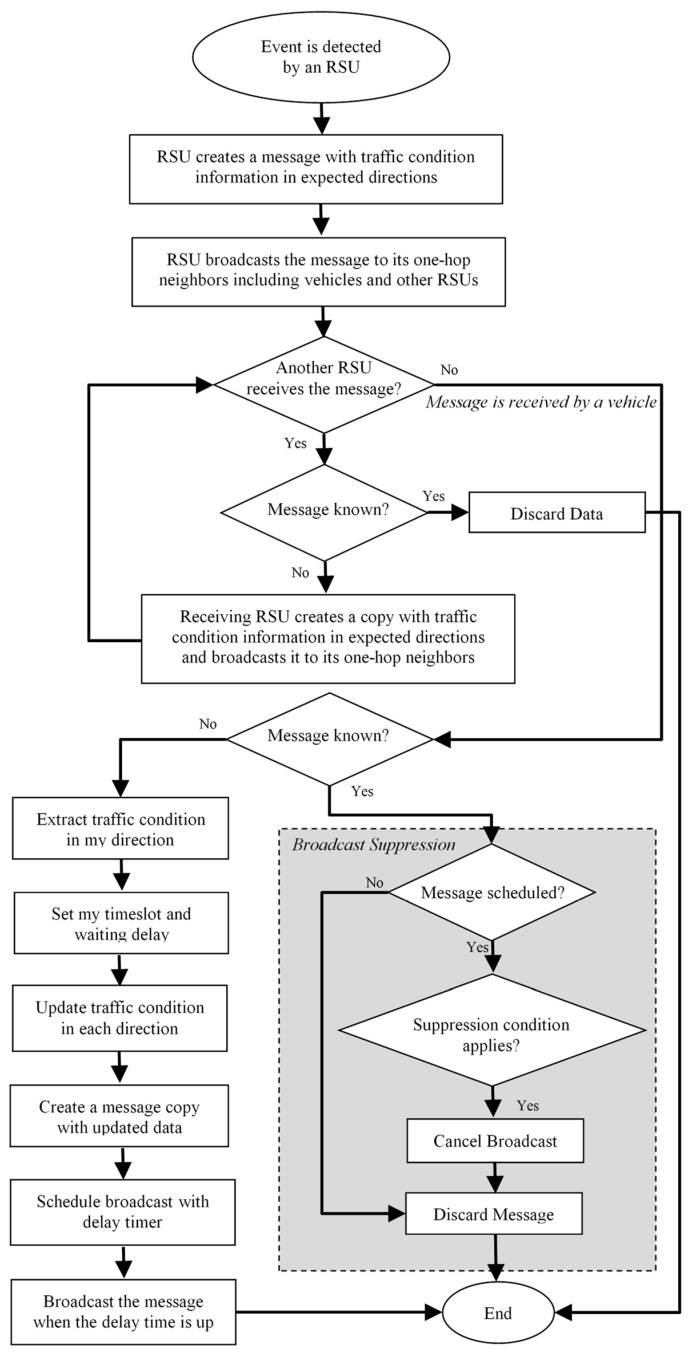
Our Proposed Edge-based Data Dissemination Protocol.

We propose to utilize the traffic condition as we described above to control data dissemination for an improved coverage, less overhead and minimized delay. Whenever a traffic event is detected, the detecting edge node initiates a message and attaches traffic condition information to it. Then it broadcasts it to its neighboring vehicles and other edges as shown in Figure 3. Each edge can predict the required traffic information to share with neighboring vehicles according to possible forwarding directions. Receiving vehicles can then accommodate their broadcasting suppression delay according to the traffic condition in their specific direction.

Receiving vehicles utilize the edge estimation of the traffic condition factor *CF* according to Equation (2), which is utilized to set the total number of timeslots *n*. This number is computed by receiving vehicles using the following linear equation:

(4) n=CF×(−m+1)+m

where *m* is the maximum number of timeslots that is set as follows:

(5) m=Rw

where *R* is the transmission radio range, and *w* is a distance parameter we set by adding the default length of a vehicle to the safety distance assumed between two vehicles. A receiving vehicle assumes n timeslots with equal distances along the transmission range, and then it determines the slot number *S_k_* it belongs to using its own location information as follows:

(6) $$S_{k}=\lfloor \left(1-\frac{\min\left(d,R\right)}{R}\right)\times n\rfloor$$

Setting the total number of timeslots n according to *CF* allows for setting more slots under low speeds, where traffic density is high, such that few vehicles participate in message forwarding, and consequently, the broadcasting overhead is decreased. On the other hand, fewer timeslots are set at high speeds, where traffic density is low, such that the transmission delay is not affected. Sharing *CF* as detected by the edge allows for setting the same number of slots *n* in each direction among all receiving vehicles. 

Waiting delays are calculated by each receiving vehicle according to its slot number *S_k_*. The delay at a certain timeslot *k* in direction *j* is computed as:

(7) $$d_{kj}=S_{k}\times \tau +\Delta_{j}$$

where *τ* is the minimum one-hop delay, which is the medium access delay added to the propagation delay. *k* is an integer between 0 and *n*, and Δ*_j_* is a random delay added to each direction to prevent collisions among different directions.

The farthest vehicles are assigned to the earliest timeslot using (6), such that they can forward messages before other vehicles that are closer to the sender. Whenever a vehicle receives a duplicate of the same message it has already received, it cancels broadcasting the same message, if scheduled. This way, the maximum forwarding distance can be reached while unnecessary broadcasts scheduled by closer vehicles are suppressed.

In the case of forwarding via VANET, traffic condition information may not be available in the received messages. Nevertheless, relay vehicles can connect to the nearest edge to update traffic condition in each direction before disseminating data further. We assume the existence of an RSU at each intersection for data collection of incoming links. Required parameters with their weights are identified such that each link has a vector of collected sensor data for each parameter. This data is collected from the latest set of traversing vehicles. An average is computed for each parameter and the condition factor is computed as in (2). The condition factor of each link is utilized to weight it in the adjacency matrix as in (1).

### 4.5. The Integration in Delay-Tolerant Applications

Our protocol is convenient for vehicular delay-tolerant applications. The key benefit that it offers to these applications is the low-latency response, because vehicles do not have to query the cloud server by themselves. Instead of querying the cloud directly, we propose to allow vehicles to query the edge first. This way, unnecessary delays are avoided. In addition, connectivity is enhanced since vehicles with no internet connection can seek traffic information by querying the nearest edge. The edge can connect to the Internet if necessary for providing appropriate responses to requesting vehicles. Since user data can be produced and consumed locally and anonymously without the need to store it or upload it to the cloud, our proposed methodology provides an enhanced privacy for drivers on the road.

**Figure 4:**
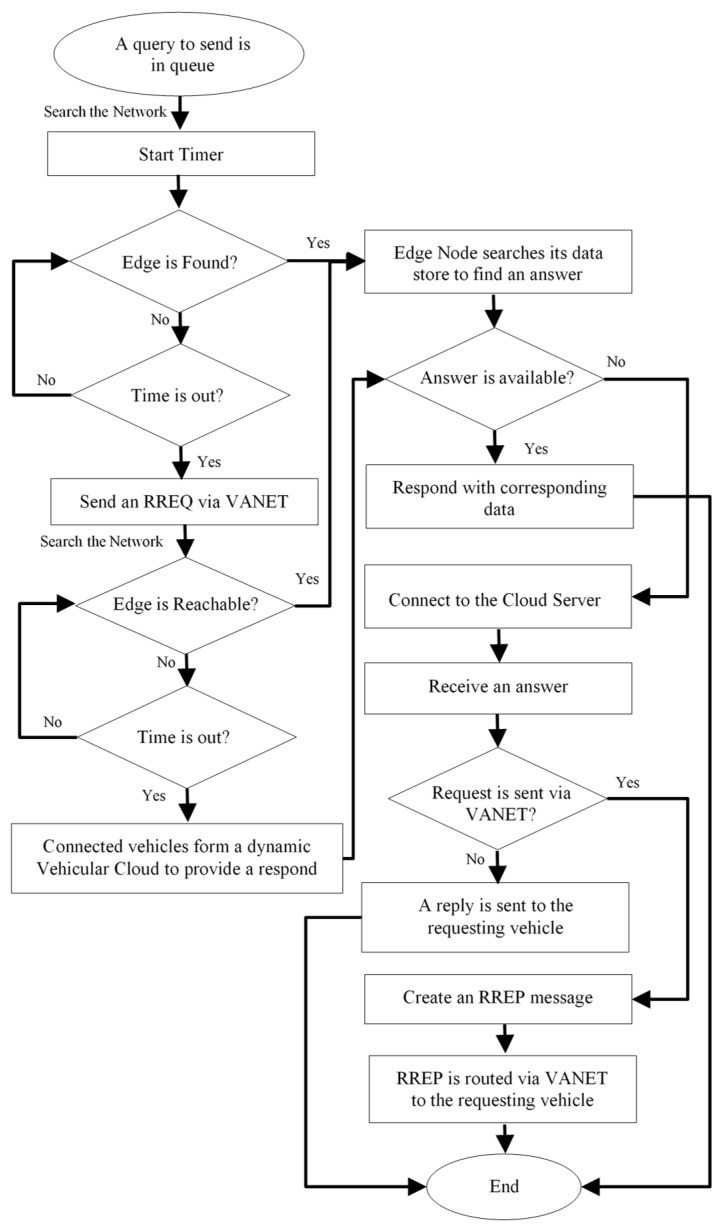
Integrating our Protocol in Delay-tolerant Applications.

An interested vehicle states its preferences for a data collection service in advance, such that it can request for traffic information on demand. The proposed integration of our protocol in delay-tolerant applications is shown in Figure 4. As the figure shows, a vehicle seeking traffic information broadcasts a query to the nearest edge node, which searches for the requested data in its local data store first. If the edge node can provide an answer, it would directly send a reply message to the requesting vehicle. Otherwise, it connects to the cloud server, receives a reply and then responds to the requesting vehicle.

If the requesting vehicle is unable to communicate with a network edge, it would create a route request (RREQ) message and broadcasts it to its one-hop neighbors. RREQ is then routed to the nearest edge by utilizing a routing protocol in a V2V communication. If the answer is available, the edge node reachable via VANET will respond with the corresponding data in a form of a route reply (RREP). RREP is routed back to the requesting vehicle via VANET. An edge node that is indirectly communicated can connect to the cloud server when an answer to the query of the requesting vehicle is not available.

In the case of sparsely connected VANET where a network edge is unreachable, we propose to utilize a dynamic vehicular cloud as described in [39]. When the RREQ is received by vehicles at the desired destination, vehicles subscribed to the same service will cooperate in providing an answer to the requesting vehicle. Whenever an internet access is required, vehicles with no internet connection can borrow these services from vehicles with an internet access via VANET. This methodology provides a generalized step-by-step procedure to request and receive traffic information on demand in a VEC environment. It merges both paradigms: VANET and MEC for a maximized connectivity. Further works would include comprehensive specifications of a VEC method for travel convenience delay-tolerant applications.

## 5. Performance Evaluation

To evaluate the performance of our proposed protocol, we provide a simulation-based implementation for vehicular data dissemination. We expect that the inclusion of traffic condition information in disseminated messages with the aid of MEC can improve the global data coverage, decrease the total dissemination overhead and minimize the dissemination delay. This improvement is expected due to the potential of utilizing traffic condition information in controlling the suppression in multi-hop delay-based broadcast. Traffic condition detection with the aid of MEC is characterized by its high accuracy, due to the global or semi-global network knowledge that is offered by edges. In contrast, VANET environment can only provide an estimated traffic condition for individual vehicles based on local data, which is sometimes misleading due to direction changes in the urban environment. In the remaining of this section, we describe our performance evaluation scenario from the following perspectives: the considered dissemination protocols, performance metrics, simulation setup and performance results.

### 5.1. Dissemination Protocols

We consider two representative protocols from the literature for performance comparison. Each protocol is simulated in VANET environment and then in MEC for comprehensive analysis. The following list describes these protocols:

- Enhanced Slotted 1-Persistance (ES1P): which is an enhanced version of the well-known Slotted 1-Persistance [40]. The enhancement is achieved by integrating the same suppression mechanism utilized in our methodology to accommodate urban vehicular environment. ES1P does not utilize traffic condition information to control the broadcast. Instead, it considers a predefined number of timeslots for setting the broadcasting delays of the receiving vehicles.
- TURBO: which is a representative example of the recent beacon-based protocols for VANETs [15]. In contrast, our methodology and ES1P utilize beacon-free data dissemination.
- ES1P-MEC: which presents the performance of ES1P with the aid of MEC.
- TURBO-MEC: which presents the performance of TURBO with the aid of MEC.

### 5.2. Performance Metrics

Performance metrics include data delivery ratio (*DR*), dissemination overhead (*DO*) and dissemination delay (*DD*). In the following, we describe each of these metrics:

- Data Delivery Ratio (*DR*); which measures the percentage of coverage. For full coverage, every vehicle should receive a copy of any sent message. At the end of the simulation, the number of messages that should be received is equal to the number of vehicles that exist whenever a message is sent, which we refer to as expected messages *M_exp_*. For every message *i*, *DR* is obtained by dividing the number of successfully received copies of the message *M_rcv_* by *M_exp_*. Assuming a total of sent messages *M_sent_*, *DR* is computed as:
  (8) $$DR=\frac{1}{M_{sent}}\overset{M_{sent}}{\underset{i=1}{\sum }}\frac{M_{rcv}}{M_{exp}}$$
- Broadcast Overhead (*BO*); which measures the average broadcast overhead per message reported by an arbitrary vehicle *j*, which is obtained by dividing the total number of duplicate messages *M_dup_*, by the number of uniquely identified messages received *M_rcv_*. Assuming *N* vehicles contributing in data dissemination, the global *BO* is computed as:
  (9) $$BO=\frac{1}{N}\overset{N}{\underset{j=1}{\sum }}\frac{M_{dup}}{M_{rcv}}$$
- Dissemination Delay (*DD*); which considers all reported delays whenever a new message is received by a vehicle to estimate an average delay for any vehicle to receive a new message. Whenever a new message *i* is received, the local delay is reported by computing the difference between the message arrival time *t_m_* and the message creation time *t*_0_ (which is stored in the message Timestamp field). Assuming the total of received messages *M_rcv_* at the end of the simulation, reported local delays are considered for computing the global average delay as follows:
  (10) $$DD=\frac{1}{M_{rcv}}\overset{M_{rcv}}{\underset{i=1}{\sum }}t_{m}-t_{0}$$

### 5.3. Simulation Setup

We utilize our proposed protocol to provide an implementation in OMNET++ [41] simulation environment. Traffic flows are generated using SUMO [42] traffic simulator. We extend the Veins [43] framework which models the vehicular communication to integrate network edges. We placed a set of edges which are assumed to collect traffic data. At the end of a predefined time cycle, each edge computes the traffic condition factor of each link it supervises, and then updates its local adjacency matrix as described in Section 4.3. These factors are utilized to control data dissemination in the vehicular network. 

**Figure 5:**
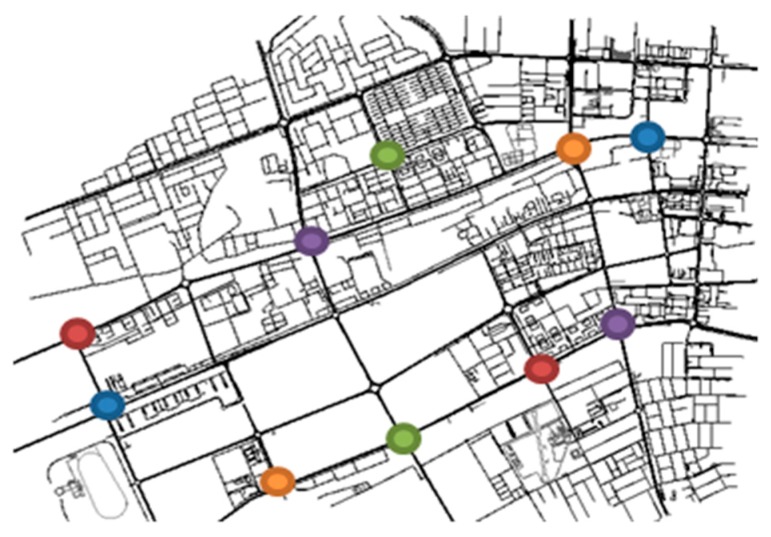
SUMO NetEdit Map.

Al Ain city map is selected for an urban road network representation. Figure 5 shows a SUMO NetEdit map with traffic generation scenarios. Each pair of colored nodes shown in the figure illustrates an Origin-Destination pair. To set the physical and the MAC layer, we utilize the implementation of IEEE 802.11p available in Veins. We rely on four traffic flow generation rates to represent different traffic scenarios ranging from low to high density. Simulation settings are summarized in Table 1.

Safety messages are generated by the network edge, which disseminates them to nearby vehicles. Receiving vehicles proceed disseminating data in multi-hop fashion via V2V communication. For performance comparison, we ran each of the selected protocols under different traffic scenarios firstly in VANET environment and then with the aid of MEC.

### 5.4. Simulation Results

**Figure 6:**
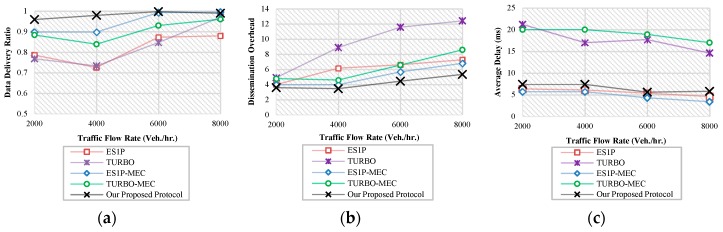
Simulation Results (**a**) Data Delivery Ratio; (**b**) Dissemination Overhead; (**c**) Dissemination Delay.

Simulation results are shown in Figure 6. As Figure 6a shows, our proposed protocol achieves almost full coverage under different traffic scenarios. The other protocols show significant improvements when evaluated under MEC architecture in terms of data delivery.

In terms of dissemination overhead, our protocol outperforms the other protocols as shown in Figure 6b. This can be explained by the utilization of traffic condition information offered by the network edge in controlling the broadcasting suppression. However, our protocol shows an increasing overhead under high traffic flow rates. This is due to the fact that a considerable percentage of messages is disseminated via V2V communication. A noticeable improvement by around 40% in the dissemination overhead is shown in TURBO-MEC compared to the original version of TURBO. This improvement is due to the role of the edge in initiating the data dissemination process on behalf of individual vehicles. This role frees the vehicles from receiving redundant data at the first hop where data is excessively disseminated.

Figure 6c shows the average dissemination delay values under different flow rates. The protocols under comparison present average delay values under MEC that are very close to those presented under VANET. Therefore, it can be concluded that the employment of a MEC architecture would not negatively affect the minimized dissemination delays of VANETs, but could significantly decrease the existing dissemination delays of cloud-based infrastructures as expected.

## 6. Issues and Challenges

Edge computing provides low-latency communication and rapid responsive speed in a distributed and dynamic manner. Edge data centers bring bandwidth-intensive content closer to the user and latency-sensitive applications closer to the data. Computing power and storage capabilities are inserted directly on the edge of the network to lower transport time and improve availability [11]. However, compared to cloud computing, edge computing provides resources with limited capacity and computation. in this section, we highlight challenges and open issues for further edge computing research in vehicular environments.

### 6.1. Deployment

The deployment of network edges within the city environment relies on both static components (such as road layout) and dynamic factors (such as accident frequency). Edge nodes are expected to be presented by equipping fixed RSUs or unmanned aerial vehicles (UAVs) with MEC servers. Many new ITS applications could be enabled by utilizing automated UAVs to improve traffic safety and enhance travel comfort. Before that, there are some serious issues to address including limited energy, processing capabilities, and signal transmission range [34]. Taking into account the technology revolution in the past decades, there is a great potential that these limitations in UAV utilization will be resolved in the near future.

### 6.2. Programmability

In cloud computing, users program their code and deploy them on the cloud, where the cloud provider is in charge to decide where the computing is conducted. The program is usually written in a certain programing language and compiled for a certain platform. However, in edge computing, computation is offloaded from the cloud, and the edge nodes are heterogeneous in most cases [28]. In the case of vehicular environments, edge nodes are associated with the existing or preplanned road infrastructure. Therefore, application designers can overcome the programmability challenge by identifying edge nodes in advance, with the objective of providing applications that may be deployed in the MEC paradigm according to the road network infrastructure.

### 6.3. Quality of Service

In the expected VEC environment, vehicles would continuously upload their local sensor data to the nearest edge. To this end, power generation and consumption at the edge should be taken into careful consideration to avoid quality of service (QoS) loss and service disruptions. In [44], the viability of solar-powered road side units (SRSU) is shown. Each SRSU is powered by solar panels with battery. It consists of small base stations in addition to a MEC server. An SRSU should allocate sufficient computational resources to the vehicles within its coverage. When the power demand of SRSU is higher than the solar generation or the energy of the battery, communication and computation resource allocation are rearranged to reduce power consumption.

In smart cities, different scenarios would require significant QoS improvement to handle temporal excessive traffic loads during public events, unexpected weather conditions, or extreme traffic congestions. In these scenarios, the employment of additional temporal RSUs that are carried by unmanned aerial vehicles (UAVs) will provide the additional required infrastructure for supporting traffic applications. UAVs can be dynamically deployed to act as mobile edges in accordance with traffic events and congestion conditions. Another idea for enhancing QoS under different traffic scenarios is proposed in [45], where the authors suggested to utilize the vehicles themselves as the infrastructures for communication and computation. A further research effort is required for enhancing and managing QoS in vehicular networks, with the consideration of a heterogeneous edge-based environment.

### 6.4. Privacy and Security

Privacy and security protection are the most important services to provide at the edge. Private information can be learned from the sensed data if the vehicle is deployed with the IoT. For example, a vehicle subscribing to IoT services via ITS can be tracked by reading its sensor data. That’s why supporting services without harming privacy is a challenging issue. Nevertheless, the edge seems to have the potential of supporting privacy compared to cloud computing. Instead of publishing user data to the cloud, processing is performed at the edge, and further cloud-based computations can rely only on aggregated data. Privacy and security challenges in edge computing include remote data access, remote computation, and context-aware computing [46].

In the context of smart cities, different ITS applications would require vehicle drivers to give access to some of the potentially private local data to untrusted vehicles which are trying to connect as edges. Efficient trust management systems along with data isolation mechanisms can effectively increase security at the edge. An attempt to ensure a secure collaboration in an edge computing environment is proposed in [47], however, a preliminary generalized framework is provided, which does not consider the challenges of vehicular networks in particular. In [48], an improved anonymous authentication scheme is proposed for vehicular networks based on secure multi-party computation theory and privacy protection protocol. The proposed scheme was theoretically analyzed, but with no evaluation in realistic scenarios. In conclusion, security and privacy are still two open issues for further research in the context of VEC.

## 7. Conclusions

Big traffic data would bring serious challenges to VANETs because of poor connectivity, less scalability, and less intelligence. Serving VANETs with cloud computing services may cause serious issues when the cloud servers are far away from the mobile vehicles due to capacity limitation and delay fluctuation of the transmission on the backbone networks. Mobile edge computing (MEC) is a promising solution to push different types of services to the edge of the radio network. This way, the computing capabilities can be offered at the edge of the mobile network, closer to mobile vehicular terminals, with low latency, high bandwidth, and real-time access to network information via traffic safety and convenience applications. 

In this paper, we explore the state of the art of integrating MEC in vehicular environments, which would allow the evolution of the next generation ITS in smart cities, and move the computation in conventional VANETs to the edge for an improved performance. Moreover, we propose an edge-based data dissemination protocol for vehicular networks to provide better connectivity, improved resource utilization, less communication overhead and minimized latency. Furthermore, we evaluate the performance of our protocol in terms of data delivery, dissemination overhead and delay. A further work would include the evaluation of MEC in more sophisticated scenarios, which could employ the collected data in different types of applications. We are currently working on a comprehensive middleware solution for facilitating different types of communications between the sensing layer and the cloud server in an edge-based vehicular environment.

## Figures and Tables

**Table 1:** Simulation Settings.

**Physical Layer**	Frequency Band	5.9 GHz
	Bandwidth	10 MHz
	Radio Range	~360 m
**MAC Layer**	MAC Bit Rate	6 Mbps
	Mac Delay	20 ms
	Data Frequency	5 Hz
**Scenario**	Urban Area	Al Ain city
	Area of Interest	6500 × 4000 m
	Max. speed	100
	Message Size	100 byte
	# of Messages	10
	Simulation time	900 s
	Number of runs	5
	Confidence Level	95%
	Traffic Flow Rates	{2000, 4000, 6000, 8000} Vehicle/hr.
